# Patterns of subjective well-being and psychopathology trajectories in adolescence: a population-based cohort study

**DOI:** 10.1017/S0033291726104243

**Published:** 2026-05-25

**Authors:** Akito Uno, Daiki Nagaoka, Rin Minami, Riki Tanaka, Yutaka Sawai, Ayako Okuma, Nanami Tomoshige, Satoshi Yamaguchi, Syudo Yamasaki, Mitsuhiro Miyashita, Atsushi Nishida, Shuntaro Ando, Kiyoto Kasai

**Affiliations:** 1 University of Tokyo: Tokyo Daigaku, Japan; 2 Tokyo Metropolitan Institute of Medical Science, Japan; 3 Yotsuba Kokoro Clinic, Japan

**Keywords:** adolescence, longitudinal trajectory, prospective birth cohort study, psychopathology, subjective well-being

## Abstract

**Background:**

Mental health encompasses subjective well-being (SWB) as well as psychopathology (PP). SWB and PP are related but separate domains: some individuals experience high SWB despite high PP, and others experience low SWB despite minimal PP. Given substantial and heterogeneous developmental changes in both domains during adolescence, examining their co-developmental trajectories may clarify how they integrate across this period.

**Methods:**

Using data from the Tokyo Teen Cohort (*N* = 2994), a population-based prospective birth cohort study, we conducted parallel process latent class growth analysis to cluster SWB–PP trajectories at ages 10, 12, 14, and 16 years. We then investigated various sociodemographic, individual, familial, and socioenvironmental correlates for each class.

**Results:**

We identified four distinct classes: high SWB–low PP (55.0%), high SWB–mid PP (20.2%), low SWB–mid PP (17.0%), and mid SWB–high PP (7.7%). SWB declined from ages 10 to 16 years across all four classes. Lower PP did not necessarily correspond to higher SWB, and in some pairs of classes, the relationship between SWB and PP levels was reversed. When comparing the two classes with moderate PP, higher aspirations, more prosocial behavior, and better interpersonal relationships were associated with the high SWB class. In contrast, being female and having a higher household income were associated with the low SWB class.

**Conclusions:**

Discrepant SWB–PP trajectories suggest characteristic patterns of developmental integration between these domains during adolescence. Considering their interplay may complement domain-specific approaches and inform psychosocial supports aimed at maintaining SWB even in the presence of PP.

## Introduction

Adolescence is a distinct developmental period marked by profound physical and psychosocial reorganization, during which mental health may have an enduring impact across the life course (Blakemore & Mills, 2014; Dahl, Allen, Wilbrecht, & Suleiman, 2018; Patton et al., 2016). The World Health Organization defines health as ‘a state of complete physical, mental and social wellbeing and not merely the absence of disease or infirmity’ (World Health Organization, 1948), highlighting that mental health is not just a matter of psychopathology (PP). In particular, the importance of focusing on subjective well-being (SWB), a person’s own evaluations of his or her life, has been emphasized in recent years (Huppert & Baylis, 2004; Steptoe, Deaton, & Stone, 2015). This contrasts with PP, which is evaluated based on socially shared concepts of illness (Maddux, Winstead, & Gosselin, 2024).

SWB and PP were previously thought to have a simple negative correlation, but they are now considered related yet separate domains of mental health (Westerhof & Keyes, 2010). The combination of SWB and PP can vary: some individuals experience high SWB despite high PP, while others experience low SWB despite minimal PP (Keyes, 2005; Kinderman et al., 2015). Although SWB–PP combinations can be described cross-sectionally as high or low on each domain (Keyes, 2005), their developmental patterns remain understudied in a longitudinal design.

A developmental perspective is particularly crucial in adolescence. Multiple longitudinal studies have shown that adolescent SWB declines with age (González-Carrasco et al., 2017; Liu, Mei, Tian, & Huebner, 2016; Orben, Lucas, Fuhrmann, & Kievit, 2022; Shek & Li, 2016; Winzer, Vaez, Lindberg, & Sorjonen, 2021). This decline of SWB in adolescence is the steepest in life, in contrast to smaller changes in adulthood (Orben, Lucas, Fuhrmann, & Kievit, 2022). On the other hand, the incidence of many mental disorders increases from early adolescence and peaks in mid-adolescence (McGrath et al., 2023). During this period, PP is often more temporally transient and fluid than in adulthood (Patel et al., 2018). In addition to these overall trends, previous studies have identified heterogeneous developmental patterns of both SWB and PP (Herke, Rathmann, & Richter, 2019; Mei et al., 2025; Rice et al., 2019; Speyer et al., 2022; Xu et al., 2022). However, given the variety of SWB-PP combinations, it remains unclear how these domains co-develop during adolescence.

While the enhancement of SWB has been discussed in positive psychology and the alleviation of PP has been discussed in clinical psychology/psychiatry (Keyes & Martin, 2017; Seligman & Csikszentmihalyi, 2000), recent epidemiological studies have found that SWB and PP are only moderately to weakly associated and each has specific correlates (Lereya, Patalay, & Deighton, 2022; Patalay & Fitzsimons, 2016). Beyond domain-specific approaches, patterns of their co-development may provide complementary insights into adolescent mental health. In the present study, we perform parallel-process clustering of SWB–PP trajectories in a population-based adolescent cohort. We then investigate various sociodemographic, individual, familial, and socioenvironmental correlates for each class. We hypothesize that classes characterized by discrepancies between SWB and PP trajectories will be identified (for example, high SWB despite high PP or low SWB despite low PP). Such discrepancies may reflect characteristic patterns of developmental integration between SWB and PP during adolescence. Examining factors associated with these classes may therefore provide insight into processes shaping adolescent mental health.

## Methods

### Study design and participants

We utilized data from the Tokyo Teen Cohort (TTC) study (Ando et al., 2019). TTC is a prospective birth cohort study of the general population that aims to examine mental and physical development during adolescence. Children born between September 2002 and August 2004 in three municipalities in Tokyo (Setagaya-ku, Mitaka-shi, and Chofu-shi) were randomly selected from the resident registers. This study used data from four waves of data collection at ages 10, 12, 14, and 16 years, conducted from October 2012 to September 2021. A total of 3171 adolescents at age 10 years, 3007 at age 12 years (follow-up rate 95%), 2665 at age 14 years (84%), and 2616 at age 16 years (83%) participated in the study. Trained examiners visited participants’ homes at each time point and administered self-report questionnaires to the child and the primary caregiver. At the first visit, written informed consent was obtained from the parents. Participants who were evaluated for both SWB and PP at least two or more timepoints were included in this study. See Supplementary Figure S1 for the flowchart of participant selection. Data were analyzed from August 2023 to May 2024. TTC is a joint study conducted by the Tokyo Metropolitan Institute of Medical Science (approval number: 12-35), the University of Tokyo (10057), and the Graduate University for Advanced Studies (2012002), and was approved by the ethics committees at each institution. This study followed the Strengthening the Reporting of Observational Studies in Epidemiology reporting guideline (Supplementary Table S1).

### Measures

#### Subjective well-being

Subjective well-being was assessed at ages 10, 12, 14, and 16 years, using the self-reported World Health Organization Well-being Index (WHO-5). A systematic review showed its validity in various countries and populations, including adolescents (Topp, Østergaard, Søndergaard, & Bech, 2015). The WHO-5 consists of five statements: ‘I have felt cheerful and in good spirits’, ‘I have felt calm and relaxed’, ‘I have felt active and vigorous’, ‘I woke up feeling fresh and rested’, and ‘My daily life has been filled with things that interest me’. Participants rated their well-being over the past 2 weeks on a 6-point Likert scale ranging from 0 (at no time) to 5 (all of the time). The raw scores, ranging from 0 to 25, were multiplied by 4, with 100 representing the highest possible well-being.

#### Psychopathology

Psychopathology was assessed at ages 10, 12, 14, and 16 years, using the caregiver-reported Strengths and Difficulties Questionnaire (SDQ) total difficulties score for 4–17-year-olds (Goodman, 1997, 2001). The SDQ is a validated psychological scale used worldwide, including with Japanese school children and adolescents (Matsuishi et al., 2008). The SDQ consists of 25 items, rated on a 3-point Likert scale ranging from 0 (not true) to 2 (certainly true). The total difficulties score is calculated by summing the subscale scores for emotional symptoms, conduct problems, hyperactivity-inattention, and peer problems. Each subscale score is based on five items, resulting in a total difficulties score ranging from 0 to 40. A higher score indicates more severe psychopathology.

#### Potential correlates

Based on previous studies examining adolescent SWB and PP (Kinderman et al., 2015; Lereya, Patalay, & Deighton, 2022; Lynch, Sunderland, Newton, & Chapman, 2021; Patalay & Fitzsimons, 2016; Patalay & Fitzsimons, 2018; Yoon, Eisenstadt, Lereya, & Deighton, 2022), the following variables were considered as potential correlates in this study: sociodemographic factors (sex, primary caregiver, ethnicity, annual household income, educational background of mother/father, house ownership, and employment of mother/father), individual factors (pubertal stage, intelligence quotient, help-seeking intention, aspirations, self-control, prosocial behavior, experience of kindergarten/elementary school entrance exam, tutoring school [private lessons outside of school], chronic health problems, special needs, and overweight), familial factors (separation from mother/father, number of siblings, elder sibling, frequency of meeting grandparent, smoker in household, life satisfaction of caregiver, chronic health problems of mother/father, cooperative spouse in childcare, satisfied with family, frequency of talking with caregiver, want to be like mother/father, caregiver involvement in future career preferences, and caregiver life plan is stability-oriented), and socioenvironmental factors (like school, social relationships outside school, parks or playgrounds in neighborhood, friends or relatives live in neighborhood, good neighborhood for childcare, and neighborhood cohesion and trust). All variables were measured by self-report or caregiver-report questionnaires when the adolescents were 10 years old. Detailed information about each variable is shown in Supplementary Table S2.

### Statistical analysis

#### Parallel process latent class growth analysis

To cluster trajectories of SWB and PP simultaneously, we conducted parallel process latent class growth analysis (LCGA). We standardized the scores of SWB and PP to adjust for the different scale ranges. Parallel process LCGA estimates a concise summary of a continuous distribution and does not allow for within-class variation. Therefore, we fixed the factor variances and covariances. Our models assumed linear or quadratic growth, and we allowed the residual variance to vary across measurement points. We created the models through optimization using the expectation maximization algorithm with 1000 random starting values. We increased the number of classes to 6. We employed maximum likelihood with robust standard errors as the estimator. Model selection primarily relied on the Lo–Mendell–Rubin test, which assesses whether the *k*-class model is superior to the *k*-1 class model (significance level set at two-sided *p* < 0.05). We also considered Akaike information criterion, Bayesian information criterion (BIC), sample size-adjusted BIC, entropy, size of the smallest class, and average posterior probability of assignment. Missing values were handled using the full information maximum likelihood (FIML) method under the assumption of missing at random, given the observed data, because FIML incorporates partially observed longitudinal outcomes directly into the likelihood function when estimating the mixture model. To make interpretation easier, we assigned names to the identified trajectory patterns. Mplus Version 8.8 was used for these analyses (Muthén & Muthén, 2017). We followed the Guidelines for Reporting on Latent Trajectory Studies (van de Schoot et al., 2017) (Supplementary Table S3).

#### Regression analysis

We examined the correlates of classes identified by parallel process LCGA using two types of regression analysis. All correlates were included in the model as independent variables, except those with a conservative variance inflation factor (VIF) cutoff >3.0 to avoid multicollinearity. Continuous variables were standardized. First, we conducted a multinomial logistic regression analysis to examine correlates of membership in each SWB–PP trajectory class relative to the reference class (the class with the largest proportion). The significance level was set at two-sided *p* < 0.05. Second, we conducted a pairwise logistic regression analysis examining membership in pairs of SWB–PP trajectory classes based on theoretical interest in the discrepancy between SWB and PP. Pairs with similar levels of SWB or PP but different levels of the other were selected. In cases where multiple comparisons were made, we planned to adjust the significance levels using a Bonferroni correction. Missing values were handled using multiple imputation methods with the mice R package (van Buuren & Groothuis-Oudshoorn, 2011) under the assumption that the data were missing at random because several correlates had missing values and multiple imputation allowed retention of the full analytic sample, thereby reducing potential selection bias. The imputation procedure included all potential correlates and outcome variables. We created 100 data sets and combined the estimates according to Rubin’s rules. R version 4.3.2 was used for these analyses (R Core Team, 2024).

#### Sensitivity analysis

To assess the robustness of our parallel process LCGA model, we performed a series of sensitivity analyses. These included refitting the model under the assumption of fixed residual variances across measurement points, refitting the model using multiple imputation to handle missing values, and refitting the model in participants with complete WHO-5 and SDQ total difficulties scores.

## Results

A total of 2994 adolescents were included in this study; 1407 participants (47.0%) were female. The primary caregiver was their mother in most cases (2949/2994 [98.5%]). Annual household income of the participants was 0–2.99 million yen for 4.6% (132/2880), 3–5.99 million yen for 25.0% (719/2884), 6–9.99 million yen for 40.6% (1170/2880), and more than 10 million yen for 29.8% (859/2880). The WHO-5 scores had missing values of 0.4% (12/2994) at age 10 years, 4.7% (141/2994) at age 12 years, 19.4% (582/2994) at age 14 years, and 24.2% (724/2994) at age 16 years. The SDQ total difficulties scores had missing values of 1.1% (32/2994) at age 10 years, 3.0% (90/2994) at age 12 years, 14.7% (440/2994) at age 14 years, and 18.9% (566/2994) at age 16 years. (Table 1) Cross-sectional correlation coefficients of WHO-5 scores and SDQ total difficulties scores ranged from −0.18 to −0.24 (Supplementary Figure S2).

**Table 1.** Baseline characteristics of the study population

Characteristic	Age (years)			
	10	12	14	16
Female, *N*/Total *N* (%)	1407/2994 (47.0%)	NA	NA	NA
*Primary caregiver, N/Total N (%)*				
Mother	2949/2994 (98.5%)	NA	NA	NA
Father	43/2994 (1.4%)	NA	NA	NA
Other	2/2994 (0.1%)	NA	NA	NA
Ethnic minority, *N*/Total *N* (%)	67/2844 (2.4%)	NA	NA	NA
*Annual household income, N/Total N (%)*				
0–2.99 million yen	132/2880 (4.6%)	NA	NA	NA
3–5.99 million yen	719/2880 (25.0%)	NA	NA	NA
6–9.99 million yen	1170/2880 (40.6%)	NA	NA	NA
More than 10 million yen	859/2880 (29.8%)	NA	NA	NA
*Educational background of mother, N/Total N (%)*				
High school or less	492/2972 (16.6%)	NA	NA	NA
Vocational school or two-year college	1312/2972 (44.1%)	NA	NA	NA
4-year university	1067/2972 (35.9%)	NA	NA	NA
6-year university or graduate school	101/2972 (3.4%)	NA	NA	NA
*Educational background of father, N/Total N (%)*				
High school or less	512/2855 (17.9%)	NA	NA	NA
Vocational school or 2-year college	385/2855 (13.5%)	NA	NA	NA
4-year university	1601/2855 (56.1%)	NA	NA	NA
6-year university or graduate school	357/2855 (12.5%)	NA	NA	NA
Employment of mother, *N*/Total *N* (%)	1758/2977 (59.1%)	NA	NA	NA
Employment of father, *N*/Total *N* (%)	2809/2857 (98.3%)	NA	NA	NA
WHO–5, mean ± SD	79 ± 16	75 ± 19	70 ± 20	67 ± 21
SDQ total difficulties score, mean ± SD	8.0 ± 4.8	7.3 ± 4.8	7.4 ± 4.9	6.7 ± 4.3

**Figure 1.**
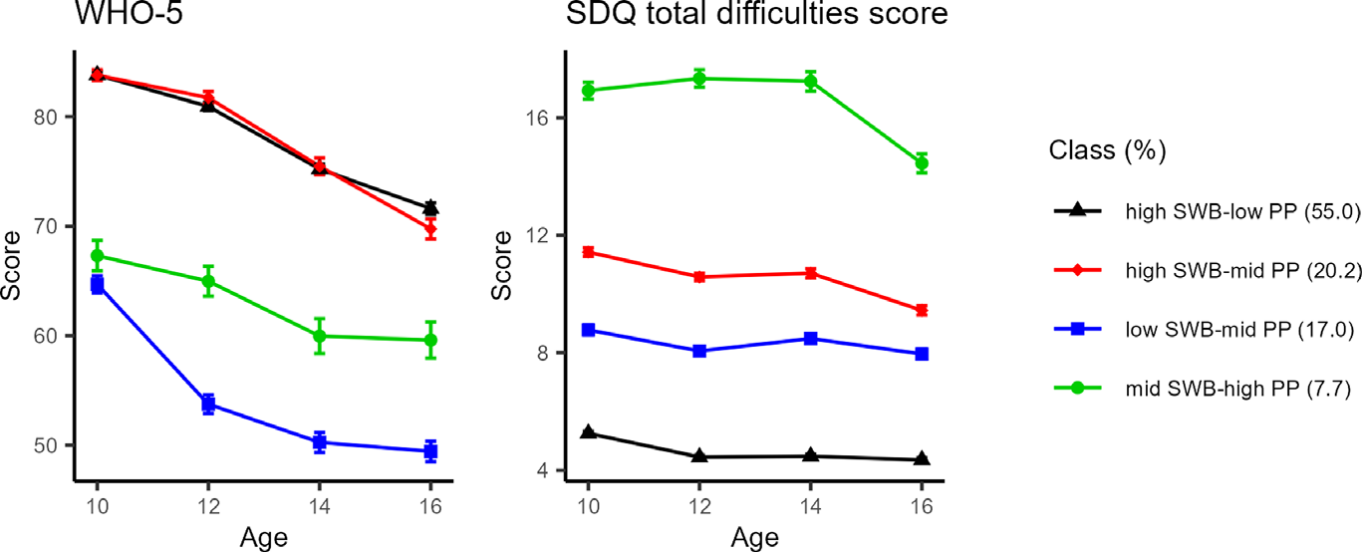
Trajectories of subjective well-being and psychopathology in the four classes identified by parallel latent class growth analysis. The points represent the mean of the WHO-5 and SDQ total difficulties score per class. Error bars indicate standard error.

Parallel process LCGA of SWB–PP trajectories supported a quadratic four-class model: high SWB–low PP (55.0% [1648/2994]), high SWB–mid PP (20.2% [606/2994]), low SWB–mid PP (17.0% [508/2994]), and mid SWB–high PP (7.7% [232/2994]) (Figure 1). The LMR test *p*-value was 0.004, the entropy was 0.80, and the average posterior probabilities of assignment were 92.4%, 82.4%, 82.3%, and 92.2% for each class (Supplementary Table S4). We presented the mean of the original scores of SWB and PP per class for ease of interpretation, because the patterns of trajectories were the same as those estimated by the standardized scores (Supplementary Figure S3). The largest high SWB–low PP class exhibited the highest SWB. Notably, the high SWB–mid PP class also showed the highest SWB despite moderate PP. In contrast, the class with the highest PP showed moderate SWB (mid SWB–high PP), while the lowest SWB was observed in the class with moderate PP (low SWB–mid PP). There was one pair with similar levels of PP but different levels of SWB: high SWB–mid PP and low SWB–mid PP. This clustering pattern was robust across all sensitivity analyses (Supplementary Figure S4–S6). The scatter plots of SWB and PP at each time point (Supplementary Figure S7) and the descriptive statistics of the sample population by classes (Supplementary Table S5) are presented in the Supplementary Material.

**Figure 2.**
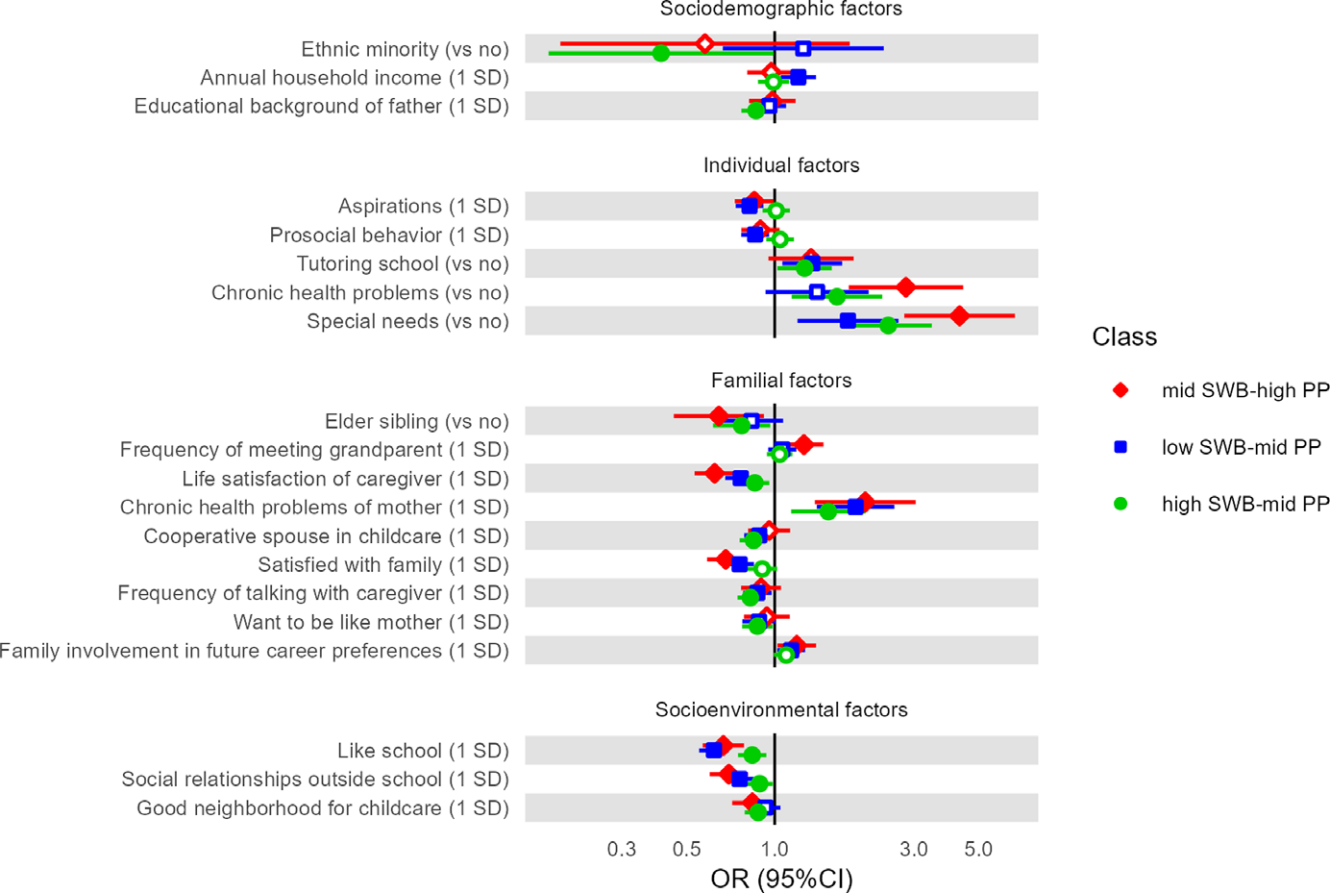
Multinomial logistic regression analysis to examine correlates of each class membership. This figure displays the results of a multinomial logistic regression that examined the correlates of class membership and their effect sizes. The outcome variable was membership to each class identified by parallel process LCGA, in which the largest class (high SWB–low PP: 55%) was treated as a reference. Filled markers represent significant OR estimates (*p* < 0.05), while blank markers indicate nonsignificant estimates. Several correlates were adjusted as independent variables in the model, but are not shown, as they did not significantly correlate with class membership. The full result is shown in Supplementary Table S7.

**Figure 3.**
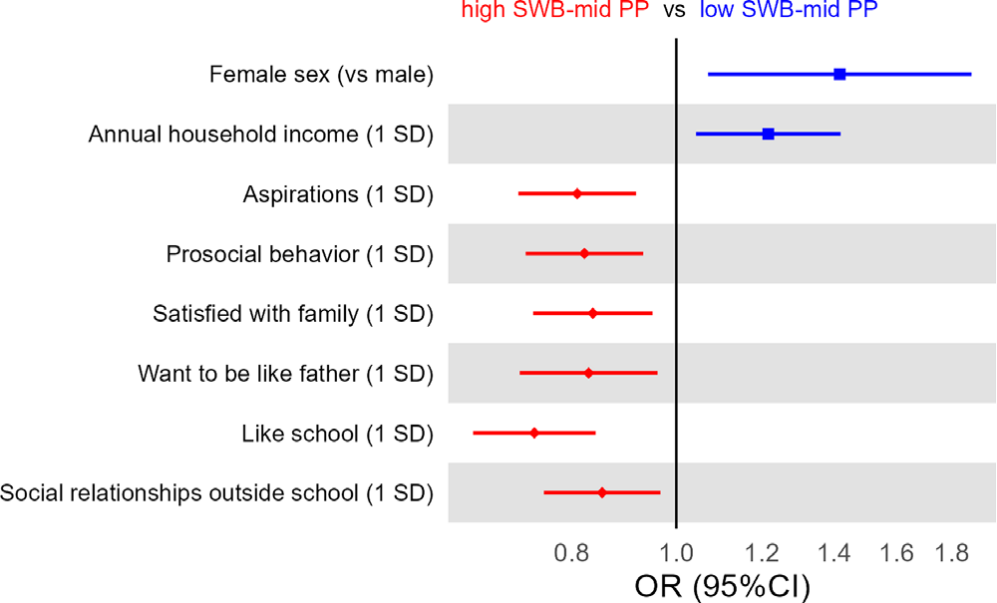
Pairwise logistic regression analysis examining correlates of membership in high SWB–mid PP or low SWB–mid PP. This figure displays the results of a pairwise logistic regression comparing high SWB–mid PP and low SWB–mid PP, in which OR > 1 correlates with membership to low SWB–mid PP and OR < 1 correlates with membership to high SWB–mid PP. Several correlates were adjusted as independent variables in the model, but are not shown, as they did not significantly correlate with class membership. The full result is shown in Supplementary Table S8.

In the subsequent regression analysis, variables with VIFs >3.0 were excluded: primary caregiver, employment of mother/father, house ownership, help-seeking intention, parks or playgrounds in the neighborhood, and friends or relatives living in the neighborhood (Supplementary Table S6). Multinomial logistic regression analysis, using the largest class (high SWB–low PP) as a reference, revealed some common correlates for high SWB–mid PP, low SWB–mid PP, and mid SWB–high PP (special needs, lower life satisfaction of caregiver, chronic health problems of mother, like school, and social relationships outside school). There were also some specific correlates for high SWB–mid PP (ethnic minority, lower educational background of father, and do not want to be like mother), low SWB–mid PP (higher annual household income and less prosocial behavior), and mid SWB–high PP (frequently meet grandparent) (Figure 2). Logistic regression analysis, as a planned pairwise comparison examining the discrepancy between SWB and PP, was performed for one pair of classes; thus, the Bonferroni correction was not adopted. In a comparison between the two classes with mid PP, higher aspirations, more prosocial behavior, satisfied with family, want to be like father, like school, and more social relationships outside school were associated with the high SWB class, while being female and having a higher household income were associated with the low SWB class (Figure 3).

## Discussion

To our knowledge, this is the first study to examine the co-developmental patterns of SWB and PP trajectories during adolescence in a general population. Parallel process LCGA identified four distinct patterns of SWB–PP trajectories. While SWB declined across all classes, ~20% of adolescents maintained high SWB despite moderate PP (high SWB–mid PP). Notably, another class exhibited the lowest SWB despite not having high PP (low SWB–mid PP). Each class was characterized by specific sociodemographic, individual, familial, and socioenvironmental correlates.

All classes showed a decline in SWB from ages 10 to 16 years, in line with global trends (González-Carrasco et al., 2017; Liu, Mei, Tian, & Huebner, 2016; Orben, Lucas, Fuhrmann, & Kievit, 2022; Shek & Li, 2016; Winzer, Vaez, Lindberg, & Sorjonen, 2021). This decline exceeds 10 points on the WHO-5 and may represent a clinically meaningful change (Topp, Østergaard, Søndergaard, & Bech, 2015). Two explanations may account for this universal decline. First, adolescence involves profound physical and psychosocial changes, as well as developmental challenges such as the quest for independence and autonomy, which may complicate life conditions (Christie & Viner, 2005; Orben, Lucas, Fuhrmann, & Kievit, 2022; Shek & Li, 2016). Second, as social cognition develops through peer relationships (Kilford, Garrett, & Blakemore, 2016), adolescents may begin to evaluate their own lives using more competitive criteria (Orben, Lucas, Fuhrmann, & Kievit, 2022; Shek & Li, 2016). Lower PP did not necessarily correspond to higher SWB, and in some cases, the relationship between SWB and PP levels was reversed (high SWB–mid PP/low SWB–mid PP and low SWB–mid PP/mid SWB–high PP). Consistent with previous cross-sectional findings (Lereya, Patalay, & Deighton, 2022; Patalay & Fitzsimons, 2016; Westerhof & Keyes, 2010), this longitudinal study further revealed that SWB and PP were related but separate domains from an adolescent developmental perspective.

In the mid SWB–high PP and low SWB–mid PP classes, SWB levels were lower than in the other two classes. While the lower SWB in the mid SWB–high PP class may reflect its higher PP, SWB was the lowest in the low SWB–mid PP class despite only moderate PP. One possible explanation for this disproportionately low SWB is that some types of distress may not be perceived as PP by adolescents or their families. A previous study found that SWB and PP were only moderately correlated (*r* = −0.48) and each had specific predictors, even when both were self-rated (Lereya, Patalay, & Deighton, 2022). On the other hand, it is also possible that certain aspects of PP might not be accurately captured by caregiver-rated measures such as SDQ. Informant disparity between caregiver-rated and self-rated PP is well-known across multiple countries (De Los Reyes & Epkins, 2023; Rescorla et al., 2013). A family environment in which adolescents’ PP is not adequately recognized by their caregivers may also contribute to lower SWB.

In a comparison between the high SWB–mid PP and low SWB–mid PP classes, higher aspirations, more prosocial behavior, and better relationships in family, school, and community were associated with the class with higher SWB. In other words, even with similar levels of PP, adolescents with these factors had higher SWB than those without them. To interpret these results, the concept of personal recovery among individuals with mental illness is insightful. Personal recovery has been defined as ‘a way of living a satisfying, hopeful and contributing life even within the limitations caused by illness’ (Anthony, 1993). Its conceptual framework is known as CHIME (connectedness, hope, and optimism about the future, identity, meaning in life, and empowerment) (Leamy et al., 2011), highlighting resources for recovery in the experience of mental illness. Although our findings address PP in the general adolescent population, they align with several aspects of the CHIME framework. Adolescents experiencing PP may maintain higher SWB not only through the reduction of PP but also through the cultivation of aspirations, prosociality, and interpersonal relationships.

Being female was associated with the low SWB–mid PP class compared with the high SWB–mid PP class, consistent with the global trend of steeper decline in SWB among girls than boys during early adolescence (Marquez et al., 2024). This gender gap in SWB may partly reflect biological factors, such as earlier pubertal onset in females (Dahl, Allen, Wilbrecht, & Suleiman, 2018). Nevertheless, substantial cross-national differences in the level of this gender gap (Campbell, Bann, & Patalay, 2021) and the persistence of the association after adjusting for pubertal stage in our regression models suggest that sociocultural factors also play an important role. Gender equality remains limited in Japan, with the country ranking 118th out of 148 nations in the Global Gender Gap Index (World Economic Forum, 2025). A cross-cultural study of adolescents in Tokyo and London reported longitudinally increasing depressive symptoms among girls, with no comparable increase among boys (Knowles et al., 2025). Moreover, although adolescent suicide has traditionally been more prevalent among boys in Japan, recent reports have documented rising suicide rates among girls, suggesting that this long-standing pattern may be reversing (Narita et al., 2025). Our findings should be interpreted within these social and cultural contexts.

Higher household income was also associated with the low SWB–mid PP class compared with the high SWB–mid PP class. While this result aligns with a British epidemiological study (Patalay & Fitzsimons, 2016), associations between SWB and income vary across populations (Main, 2018; Sarriera et al., 2015). Ecological analyses of the Programme for International Student Assessment suggested that adolescents in wealthier nations tended to report lower SWB, partly attributable to academic pressure, particularly among girls (Rudolf & Bethmann, 2023). In Japan, particularly in the Tokyo metropolitan area, higher-income and more highly educated families are more likely to pursue selective school pathways, often involving competitive entrance examinations and prolonged extracurricular study (Kataoka, 2009; Ohashi, Iume, & Togo, 2023). Such academic pressure may partly explain the lower SWB observed among adolescents from higher-income households, even when PP levels are moderate.

The strengths of this study included comprehensive data collection and a high follow-up rate, which enabled clustering of co-developmental SWB–PP trajectories and examination of various correlates. Nonetheless, several limitations should be acknowledged. First, measurement bias may have influenced our findings. SWB was assessed using the self-rated WHO-5, which captures hedonic well-being but does not fully reflect eudaimonic aspects (Ryan & Deci, 2001). PP was assessed using the caregiver-rated SDQ total difficulties score, which does not distinguish among specific psychopathological domains and does not include some domains, such as psychosis. Moreover, caregiver-rated PP may differ from self-rated PP. It is possible that our results may reflect not only differences between SWB and PP but also differences in measurement constructs and informants. Second, the identified patterns of SWB–PP trajectories may not be directly generalizable to other cultural contexts. For example, Japan is often characterized as relatively collectivistic, and public stigma toward mental illness is more readily internalized by individuals (Yu et al., 2021), which may shape the association between SWB and PP. Because SWB in Japan is associated with social harmony rather than personal achievement (Uchida & Kitayama, 2009), attitudes toward PP among significant others, such as caregivers, may also influence this association. Alternative patterns may emerge in other sociocultural settings. Third, important variables such as genetics and gender identity were not available in our dataset. Therefore, biological sex reported by caregivers was used. It is worth noting that peer problems were considered as a component of the outcome variable PP (Supplementary Table S2), rather than correlates. Additionally, all correlates were evaluated at age 10 years, ignoring time-varying effects. Fourth, our parallel process LCGA model converged under the assumption of no within-class random effects. Finally, as an observational study, causal interpretation is not warranted. Estimates for each correlate depend on the variables included in the model. Future research using causal inference approaches may clarify underlying mechanisms and pathways.

## Conclusion

This study identified distinct co-developmental patterns of SWB and PP, including classes characterized by discrepancies between the two trajectories, which may reflect characteristic integration of these mental health domains during adolescence. Cultivating aspirations, prosociality, and interpersonal relationships may help maintain SWB even in the presence of PP, complementing approaches that focus separately on enhancing SWB or alleviating PP. In contrast, being female and having a higher household income were associated with the lowest SWB despite moderate PP, suggesting that sociocultural and structural contexts may influence adolescent SWB beyond levels of PP.

## Supporting information

10.1017/S0033291726104243.sm001 Uno et al. supplementary material Uno et al. supplementary material

## Supplementary material

The supplementary material for this article can be found at http://doi.org/10.1017/S0033291726104243.
