# Supplementary material for: Patterns of subjective well-being and psychopathology trajectories in adolescence: a population-based cohort study
Source: Psychol Med. 2026 May 25;56:e147. doi: 10.1017/S0033291726104243 (PMC13200148; doi:10.1017/S0033291726104243)
Supplement: Uno et al. supplementary material [file S0033291726104243sup001.docx]

**Patterns of subjective well-being and psychopathology trajectories in adolescence: A population-based cohort study**

**Supplementary material**

**Figure S1.** Flowchart of participant selection

**Table S1.** Strengthening the Reporting of Observational Studies in Epidemiology (STROBE) reporting guideline

**Table S2.** Detailed information on Measures

**Table S3.** Guidelines for Reporting on Latent Trajectory Studies (GRoLTS) Checklist

**Figure S2.** Correlations between WHO-5 scores and SDQ total difficulties scores

**Table S4.** Fit statistics of parallel process latent class growth analysis

**Figure S3.** Estimated means of the models and the observed individual trajectories

**Figure S4.** Results of the parallel latent class growth analysis under the assumption of fixed residual variance across measurement points

**Figure S5.** Results of the parallel latent class growth analysis using multiple imputations to handle missing values

**Figure S6.** Results of the parallel latent class growth analysis in participants with complete WHO-5 and SDQ total difficulties scores

**Figure S7.** Scatter plots of WHO-5 scores and SDQ total difficulties scores at each time point

**Table S5.** Descriptive statistics of the sample population both as a whole and by classes

**Table S6.** Variation inflation factor (VIF) of correlates included in multinominal regression

**Table S7.** Full result of the multinomial logistic regression analysis to examine correlates of each class membership

**Table S8.** Full result of the pairwise logistic regression analysis examining membership to high SWB-mid PP or low SWB-mid PP

**References**



**Figure S1.** Flowchart of participant selection

**Table S1.** Strengthening the Reporting of Observational Studies in Epidemiology (STROBE) reporting guideline

|  | Item No | Recommendation | Page No |
| --- | --- | --- | --- |
| **Title and abstract** | 1 | (*a*) Indicate the study’s design with a commonly used term in the title or the abstract | 1, 2 |
|  |  | (*b*) Provide in the abstract an informative and balanced summary of what was done and what was found | 2 |
| Introduction | | | |
| Background/rationale | 2 | Explain the scientific background and rationale for the investigation being reported | 3, 4 |
| Objectives | 3 | State specific objectives, including any prespecified hypotheses | 3, 4 |
| Methods | | | |
| Study design | 4 | Present key elements of study design early in the paper | 5 |
| Setting | 5 | Describe the setting, locations, and relevant dates, including periods of recruitment, exposure, follow-up, and data collection | 5 |
| Participants | 6 | (*a*) Give the eligibility criteria, and the sources and methods of selection of participants. Describe methods of follow-up | 5 |
|  |  | (*b*) For matched studies, give matching criteria and number of exposed and unexposed | - |
| Variables | 7 | Clearly define all outcomes, exposures, predictors, potential confounders, and effect modifiers. Give diagnostic criteria, if applicable | 5-7 |
| Data sources/ measurement | 8* | For each variable of interest, give sources of data and details of methods of assessment (measurement). Describe comparability of assessment methods if there is more than one group | 5-7, Table S2 |
| Bias | 9 | Describe any efforts to address potential sources of bias | 16 |
| Study size | 10 | Explain how the study size was arrived at | Figure S1 |
| Quantitative variables | 11 | Explain how quantitative variables were handled in the analyses. If applicable, describe which groupings were chosen and why | 5-7, Table S2 |
| Statistical methods | 12 | (*a*) Describe all statistical methods, including those used to control for confounding | 7-9 |
|  |  | (*b*) Describe any methods used to examine subgroups and interactions | 7-9 |
|  |  | (*c*) Explain how missing data were addressed | 7-9 |
|  |  | (*d*) If applicable, explain how loss to follow-up was addressed | 7-9 |
|  |  | (*e*) Describe any sensitivity analyses | 9 |
| Results | | |  |
| Participants | 13* | (a) Report numbers of individuals at each stage of study—eg numbers potentially eligible, examined for eligibility, confirmed eligible, included in the study, completing follow-up, and analysed | 9, 10 |
|  |  | (b) Give reasons for non-participation at each stage | 9, 10 |
|  |  | (c) Consider use of a flow diagram | Figure S1 |
| Descriptive data | 14* | (a) Give characteristics of study participants (eg demographic, clinical, social) and information on exposures and potential confounders | 9, 10,  Table 1,  Table S5 |
|  |  | (b) Indicate number of participants with missing data for each variable of interest | Table 1,  Table S5 |
|  |  | (c) Summarise follow-up time (eg, average and total amount) | - |
| Outcome data | 15* | Report numbers of outcome events or summary measures over time | 9, 10,  Table 1,  Table S5 |

| Main results | 16 | (*a*) Give unadjusted estimates and, if applicable, confounder-adjusted estimates and their precision (eg, 95% confidence interval). Make clear which confounders were adjusted for and why they were included | 10, 11 |
| --- | --- | --- | --- |
|  |  | (*b*) Report category boundaries when continuous variables were categorized | Table S2 |
|  |  | (*c*) If relevant, consider translating estimates of relative risk into absolute risk for a meaningful time period | - |
| Other analyses | 17 | Report other analyses done—eg analyses of subgroups and interactions, and sensitivity analyses | 10, 11 |
| Discussion | | | |
| Key results | 18 | Summarise key results with reference to study objectives | 11, 12 |
| Limitations | 19 | Discuss limitations of the study, taking into account sources of potential bias or imprecision. Discuss both direction and magnitude of any potential bias | 15, 16 |
| Interpretation | 20 | Give a cautious overall interpretation of results considering objectives, limitations, multiplicity of analyses, results from similar studies, and other relevant evidence | 12-15 |
| Generalisability | 21 | Discuss the generalisability (external validity) of the study results | 15, 16 |
| Other information | | | |
| Funding | 22 | Give the source of funding and the role of the funders for the present study and, if applicable, for the original study on which the present article is based | 17 |

*Give information separately for exposed and unexposed groups.

**Table S2.** Detailed information on Measures

| **Characteristic** | **Type of variable** | **Range** | **Detail** |
| --- | --- | --- | --- |
| **Sociodemographic factors** | | | |
| Female | categorical | - | Primary caregivers answered male or female when adolescents were at age 10. |
| Primary caregiver | categorical | - | The respondents as primary caregivers when adolescents were at age 10. |
| Ethnic minority | categorical | - | Primary caregivers answered the ethnicity of themselves and their partners when adolescents were at age 10. If at least one of the answers were not Japanese, adolescents were considered as ethnic minority. |
| Annual household income | continuous | 1-11 | Primary caregivers answered 1="0 to 0.99 million yen", 2="1 to 1.99 million yen", 3="2 to 2.99 million yen", 4="3 to 3.99 million yen", 5="4 to 4.99 million yen", 6="5 to 5.99 million yen", 7="6 to 6.99 million yen", 8="7 to 7.99 million yen", 9="8 to 8.99 million yen", 10="9 to 9.99 million yen" or 11="more than 10 million yen" when adolescents were at age 10. |
| Educational background of mother/father | continuous | 1-6 | Primary caregivers were asked about the educational background of themselves and their partners when adolescents were at age 10. They answered 1="elementary school", 2="junior high school", 3="high school", 4="vocational school or two-year college" 5="four-year university", or 6="six-year university or graduate school". |
| House ownership | categorical | - | Primary caregivers were asked "Do you live in your own house" and answered yes or no when adolescents were at age 10. |
| Employment of mother/father | categorical | - | Primary caregivers were asked about the employment status of themselves and their partners and answered yes or no when adolescents were at age 10. |
| **Individual factors** | | | |
| Pubertal stage | continuous | 1-5 | The Tanner stage was used to assess pubertal development[^1^](https://sciwheel.com/work/citation?ids=7359540&pre=&suf=&sa=0&dbf=0). Scores ranged from 1 (prepubertal) to 5 (fully matured). Primary caregivers rated the Tanner stage according to pubic hair (both sexes) and breast (girls) or genital (boys) development when adolescents were at age 10. The maximum of pubic hair and breast/genitals was used to represent the overall pubertal stage[^2^](https://sciwheel.com/work/citation?ids=12593255&pre=&suf=&sa=0&dbf=0). |
| Intelligence quotient (IQ) | continuous | 64.2-140.3 | Adolescents' intelligence quotient (IQ) was estimated by two subsets (Information and Picture Completion) of the Wechsler Intelligence Scale for Children (WISC-Ⅲ)[^3^](https://sciwheel.com/work/citation?ids=15772445&pre=&suf=&sa=0&dbf=0) when they were at age 10. We developed a formula (Estimated IQ = 3.3*Information + 2.0*Picture Completion + 45.6) to estimate the adolescents' IQ from the two subsets using data from 28 participants who were tested using the full version of WISC-III by expert psychologists after one year. The IQ calculated by the formula explained 78% of the variance in IQ from the full version of WISC-Ⅲ[^4,5^](https://sciwheel.com/work/citation?ids=11066107,11530982&pre=&pre=&suf=&suf=&sa=0,0&dbf=0&dbf=0). |
| Help-seeking intention | categorical | - | Adolescents were asked "If you were in the same situation as Taro, would you talk to someone about it?" and answered "talk to someone immediately" or "no one" (A sentence is presented in advance that indicates that a child named Taro is depressed). |
| Aspirations | continuous | 1-4 | Adolescents were asked "Are you looking forward to growing up?" and answered 1="no", 2="somewhat no", 3="somewhat yes", or "yes" when they were at age 10. |
| Self-control | continuous | 4-12 | Adolescents' self-control was assessed by sum of 4 item scores of forerunners of those used in the Rutter A scale[^6^](https://sciwheel.com/work/citation?ids=15371460&pre=&suf=&sa=0&dbf=0) based on the factor analysis in previous studies[^7,8^](https://sciwheel.com/work/citation?ids=16509574,16500808&pre=&pre=&suf=&suf=&sa=0,0&dbf=0&dbf=0). Primary caregivers were asked "Which statement in each group best describes this child?" when adolescents were at age 10. The answers for attitude to work were 1="A poor worker or lazy", 2="Average–works moderately well", and 3="A very hard worker". The answers for concentration were 1="Little or no power of sustained concentration", 2="Average–concentrates moderately well", or 3="One with high power of concentration". The answers for neatness in work were 1="Very untidy in work", 2="Average–moderately neat and tidy", or 3="Extremely neat and tidy in work". The answers for daydreaming were 1="Frequently daydreams", 2="Sometimes daydreams", or 3="Seldom or never daydreams". |
| Prosocial behavior | continuous | 1-4 | Adolescents were asked "Do you want to help someone in need?" and answered 1="no", 2="somewhat no", 3="somewhat yes", or "yes" when they were at age 10. |
| Experience of kindergarten entrance exam | categorical | - | Primary caregivers were asked "Did your child take the Experience of kindergarten entrance exam?" and answered "yes" or "no" when adolescents were at age 10. |
| Experience of elementary school entrance exam | categorical | - | Primary caregivers were asked "Did your child take the Experience of elementary school entrance exam?" and answered "yes" or "no" when adolescents were at age 10. |
| Tutoring school | categorical | - | Primary caregivers were asked "Does your child currently go to a tutoring school?" and answered "yes" or "no" when adolescents were at age 10. |
| Chronic health problems | categorical | - | Primary caregivers were asked "Has your child experienced any physical or mental health problems that have lasted more than one year? (Include any that are expected to last more than a year in the future)" and answered "yes" or "no" when adolescents were at age 10. |
| Special needs | categorical | - | Primary caregivers were asked "Does your child need more medical, mental health, or educational services than other children of the same age?" and answered "yes" or "no" when adolescents were at age 10. |
| Overweight | categorical | - | Based on the health examination manual from the Japan Society of School Health[^9^](https://sciwheel.com/work/citation?ids=15948255&pre=&suf=&sa=0&dbf=0), overweight was defined as being more than 20% over the standard body weight. Standard body weights were calculated from the heights at age 10 using a formula deduced for each sex of Japanese children. |
| **Familial factors** | | | |
| Separation from mother/father | categorical | - | Primary caregivers were asked "Has your child ever lived away from you (primary caregiver)/your partner for more than one year during the past two years?" and answered "yes" or "no" when adolescents were at age 10. |
| Number of siblings | continuous | - | Primary caregivers answered the number of siblings when adolescents were at age 10. |
| Elder sibling | categorical | - | Primary caregivers answered about elder siblings when adolescents were at age 10. |
| Frequency of meeting grandparent | continuous | 1-8 | Primary caregivers answered "How often does your child meet grandparents?" when adolescents were at age 10. The answers were 1="none", 2="once in 2 or 3 years", 3="once or twice a year", 4="less than once a month", 5="once or twice a month", 6="once or twice a week", 7="several times a week", or 8="every day or almost every day". If all grandparents had passed away, this variable was treated as a missing value. |
| Smoker in household | categorical | - | Primary caregivers were asked about current smoking of themselves and their partners and answered "yes" or "no" when adolescents were at age 10. If either answer was "yes", this variable was considered as "yes". |
| Life satisfaction of caregiver | continuous | 0-10 | Primary caregivers were asked "How satisfied are you with your overall life these days?" and answered on an 11-point Likert scale ranging from 0="not satisfied at all" to 10="very satisfied" when adolescents were at age 10. Responses on a Likert scale were used as continuous variables. |
| Chronic health problems of mother/father | categorical | - | Primary caregivers were asked the question "Have you/your partner experienced any physical or mental health problems that have lasted for more than one year? (This includes those that are expected to last more than one year in the future.)" and answered "yes" or "no" when adolescents were at age 10. |
| Cooperative spouse in childcare | continuous | 1-4 | Primary caregivers were asked “Is your partner helpful in times of need on childcare?” and answered 1="rarely helpful", 2="not very helpful", 3="usually helpful", or 4="always helpful" when adolescents were at age 10. |
| Satisfied with family | continuous | 0-6 | Adolescents were asked "How do you feel about your family?" and answered on a 7-point Likert scale ranging from 1="not satisfied at all" to 7="very satisfied" when they were at age 10. Responses on a Likert scale were used as continuous variables. |
| Frequency of talking with caregiver | continuous | 1-4 | Primary caregivers were asked “Do you talk with your child often?” and answered 1="sometimes do not talk for more than 1 day", 2=" always talk at least once a day", 3="talk for about 1 hour a day", or 4="talk for more than 2 hours a day" when adolescents were at age 10. |
| Want to be like mother/father | continuous | 1-4 | Adolescents were asked “Do you want to be like your mother/father in the future?” and answered 1="disagree", 2="somewhat disagree", 3="somewhat agree", or 4="agree" when they were at age 10. |
| Family involvement in future career preferences | continuous | 1-4 | Adolescents were asked "Does your family involve in your future occupation in the way you should do this or that?" and answered 1="never", 2="seldom", 3="sometimes", or 4="always" when they were at age 10. |
| Caregiver life plan is stability-oriented | continuous | 0-10 | Primary caregivers were asked "What kind of future do you want for your child?" and answered on an 11-point Likert scale ranging from 0="a challenging life despite risks" to 10="a stable life with minimal risks" when adolescents were at age 10. Responses on a Likert scale were used as continuous variables. |
| **Socioenvironmental factors** | | | |
| Like school | continuous | 1-3 | The adolescents were asked the question "Do you like school?" and answered "very much", "somewhat", or "not at all" when they were at age 10. The responses were then grouped into yes (answered "very much" or "somewhat") and no (answered "not at all"). |
| Social relationships outside school | continuous | 1-4 | Primary caregivers were asked "Does your child have opportunities to interact with adults other than family members or children of different ages in activities outside of school? (e.g., boy scouts, little league, club teams, local children’s group, church, volunteering, etc.)" and answered 1="never", 2="seldom", 3="sometimes", or 4="always" when they were at age 10. |
| Parks or playgrounds in neighborhood | categorical | - | Adolescents were asked "Are there parks or playgrounds in your neighborhood where children your age can play outside" and answered "yes" or "no" when they were at age 10. |
| Friends or relatives live in neighborhood | categorical | - | Primary caregivers were asked "Do your friends or relatives live in your neighborhood" and answered "friends live", "relatives live ", "both friends and relatives live", or "no one lives" when adolescents were at age 10. If either friends or families live, this variable was considered as "yes". |
| Good neighborhood for childcare | continuous | 1-5 | Primary caregivers were asked "Is your neighborhood good for childcare?" and answered 1="very poor", 2="poor", 3="average", 4="good", or 5="excellent" when adolescents were at age 10. |
| Neighborhood cohesion and trust | continuous | 5-25 | Neighborhood cohesion and trust was assessed as the sum of item scores derived from the social cohesion and trust subscale of the collective efficacy scale[^10^](https://sciwheel.com/work/citation?ids=743318&pre=&suf=&sa=0&dbf=0). Items are as follows: "People around here are willing to help their neighbors", "This is a close knit neighborhood", "People in this neighborhood can be trusted", "People in this neighborhood generally get along with each other", and "People in this neighborhood share the same values". Primary caregivers were asked these items and answered 1="very unlikely", 2="unlikely", 3="neither likely nor unlikely", 4="likely", or 5="very likely" when adolescents were at age 10. |

**Table S3.** Guidelines for Reporting on Latent Trajectory Studies (GRoLTS) Checklist

|  | **Checklist Item** | **Reported?** |
| --- | --- | --- |
| 1. | Is the metric of time used in the statistical model reported? | Yes |
| 2. | Is information presented about the mean and variance of time within a wave? | Yes |
| 3a. | Is the missing data mechanism reported? | Yes |
| 3c. | Is a description provided of what variables are related to attrition/missing data? | No |
| 4. | Is information about the distribution of the observed variables included? | Yes |
| 5. | Is the software mentioned? | Yes |
| 6a. | Are alternative specifications of within-class heterogeneity considered (e.g., LCGA vs. LGMM) and clearly documented? If not, was sufficient justification provided as to eliminate certain specifications from consideration? | Yes |
| 6b. | Are alternative specifications of the between-class differences in variance-covariance matrix structure considered and clearly documented? If not, was sufficient justification provided as to eliminate certain specifications from consideration? | Yes |
| 7. | Are alternative shape/functional forms of the trajectories described? | Yes |
| 8. | If covariates have been used, can analyses still be replicated? | No |
| 9. | Is information reported about the number of random start values and final iterations included? | Yes |
| 10. | Are the model comparison (and selection) tools described from a statistical perspective? | Yes |
| 11. | Are the total number of fitted models reported, including a one-class solution? | Yes |
| 12. | Are the number of cases per class reported for each model (absolute sample size, or proportion)? | Yes |
| 13. | If classification of cases in a trajectory is the goal, is entropy reported? | Yes |
| 14a. | Is a plot included with the estimated mean trajectories of the final solution? | Yes |
| 14b. | Are plots included with the estimated mean trajectories for each model? | Yes |
| 14c. | Is a plot included of the combination of estimated means of the final model and the observed individual trajectories split out for each latent class? | Yes |
| 15. | Are characteristics of the final class solution numerically described (i.e., means, SD/SE, n, CI, etc.)? | Yes |
| 16. | Are the syntax files available (either in the appendix, supplementary materials, or from the authors)? | Yes |

**Figure S2.** Correlations between WHO-5 scores and SDQ total difficulties scores
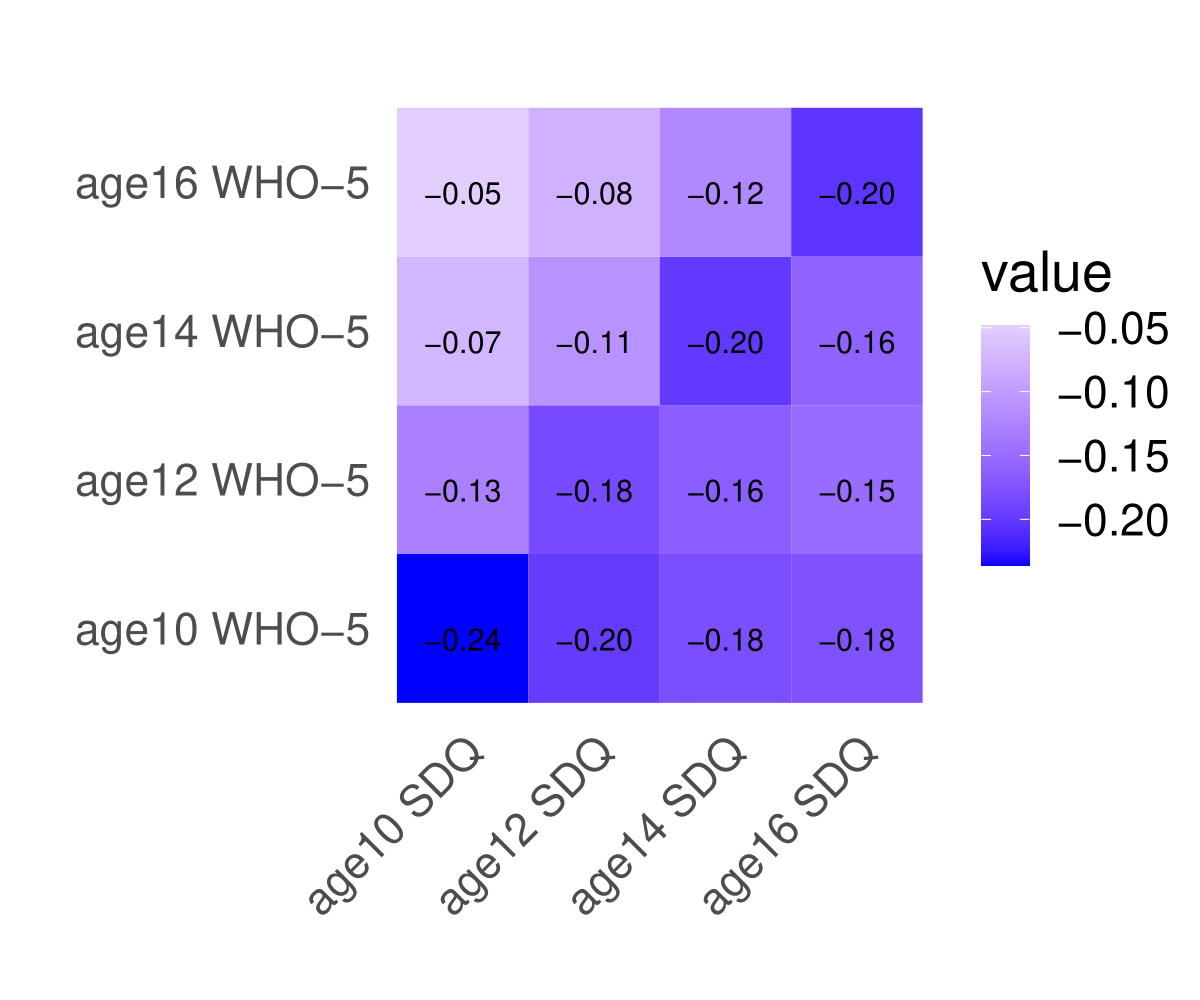


**Table S4.** Fit statistics of parallel process latent class growth analysis

| **Model** | **Number of classes** | **LL** | **AIC** | **BIC** | **aBIC** | **Entropy** | **LMR test *p* value** | **proportion per class (%)** | **APPA** |
| --- | --- | --- | --- | --- | --- | --- | --- | --- | --- |
| Linear | 1 | -29817 | 59659 | 59731 | 59693 | NA | NA | 100 | 100 |
| Linear | 2 | -27700 | 55434 | 55536 | 55482 | 0.862 | 0.000 | 74.6: 25.4 | 97.1: 92.7 |
| Linear | 3 | -27063 | 54171 | 54303 | 54233 | 0.826 | 0.000 | 58.4: 33.7: 7.9 | 93.6: 88.7: 93.1 |
| Linear | 4 | -26758 | 53570 | 53732 | 53646 | 0.793 | 0.002 | 55: 20: 17.1: 7.9 | 92.4: 82.1: 81.3: 91.7 |
| Linear | 5 | -26523 | 53110 | 53302 | 53200 | 0.758 | 0.168 | 42: 22.6: 20.9: 8.8: 5.7 | 87.4: 79.1: 82.1: 82.3: 90.3 |
| Linear | 6 | -26363 | 52800 | 53022 | 52904 | 0.767 | 0.665 | 41: 21: 20.6: 8.9: 5.7: 2.7 | 87.7: 78: 78.3: 84.2: 82.5: 90.6 |
| Quadratic | 1 | -29817 | 59662 | 59746 | 59702 | NA | NA | 100 | 100 |
| Quadratic | 2 | -27683 | 55407 | 55533 | 55466 | 0.863 | 0.000 | 74.6: 25.4 | 97.2: 92.5 |
| Quadratic | 3 | -27033 | 54122 | 54291 | 54202 | 0.828 | 0.000 | 58.5: 33.5: 7.9 | 93.8: 88.5: 93.3 |
| **Quadratic** | **4** | **-26714** | **53499** | **53709** | **53598** | **0.797** | **0.004** | **55: 20.2: 17: 7.7** | **92.4: 82.4: 82.3: 92.2** |
| Quadratic | 5 | -26470 | 53025 | 53277 | 53143 | 0.769 | 0.214 | 43.5: 22.1: 20.6: 8.7: 5 | 88.3: 79.8: 82.9: 81.9: 91 |
| Quadratic | 6 | -25796 | 51701 | 52031 | 51856 | 0.755 | 0.104 | 55: 28.2: 5.1: 4.4: 3.7: 3.6 | 86.9: 75.4: 84.7: 81: 79.9: 74.5 |

NA: not applicable, LL: log-likelihood, AIC: Akaike information criterion, BIC: Bayesian information, aBIC: simple size adjusted BIC, LMR: Lo-Mendell-Rubin, APPA: the average posterior probability of assignment


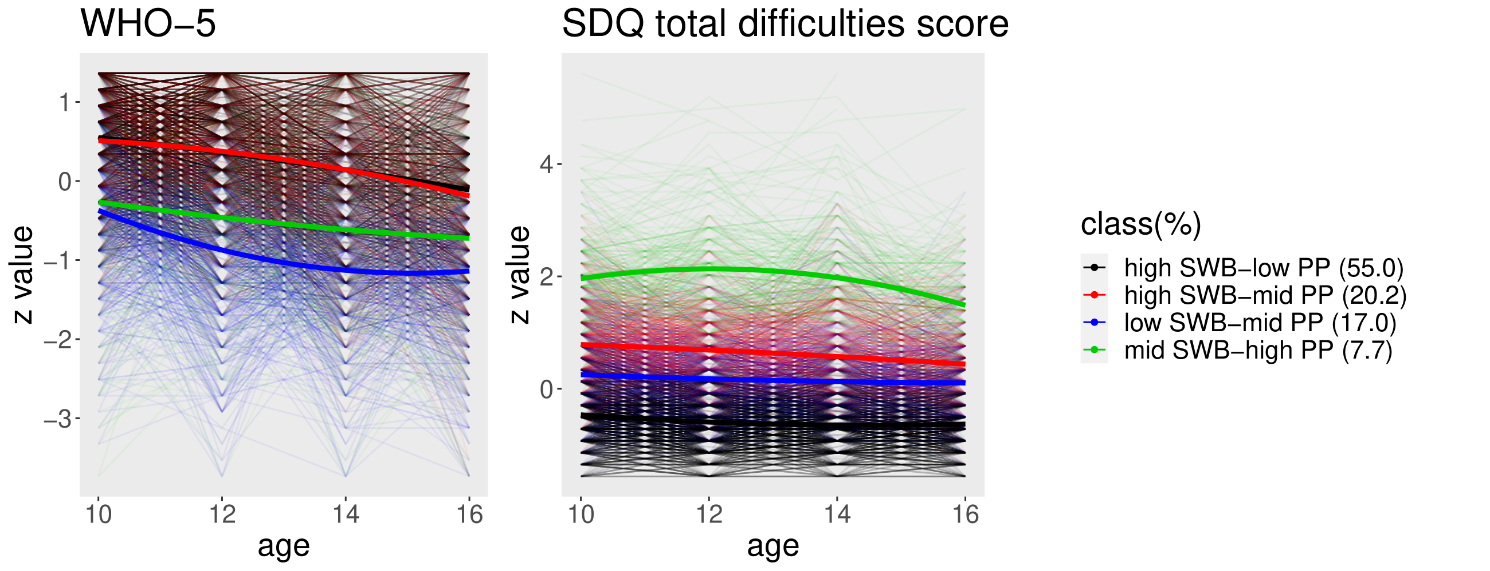


**Figure S3.** Estimated means of the models and the observed individual trajectories


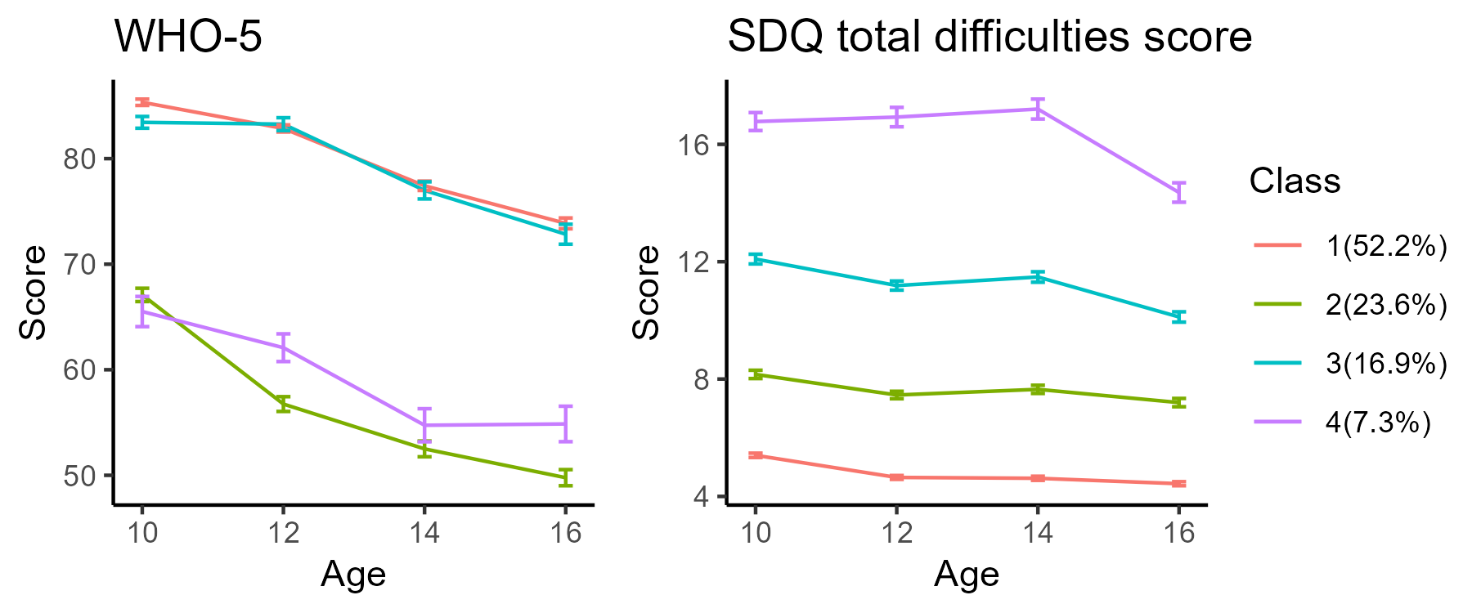


**Figure S4.** Results of the parallel latent class growth analysis under the assumption of fixed residual variance across measurement points

The points represent the mean of WHO-5 and SDQ total difficulties score per class. Error bars indicate standard error. Class numbers were assigned in order of their proportions.

**
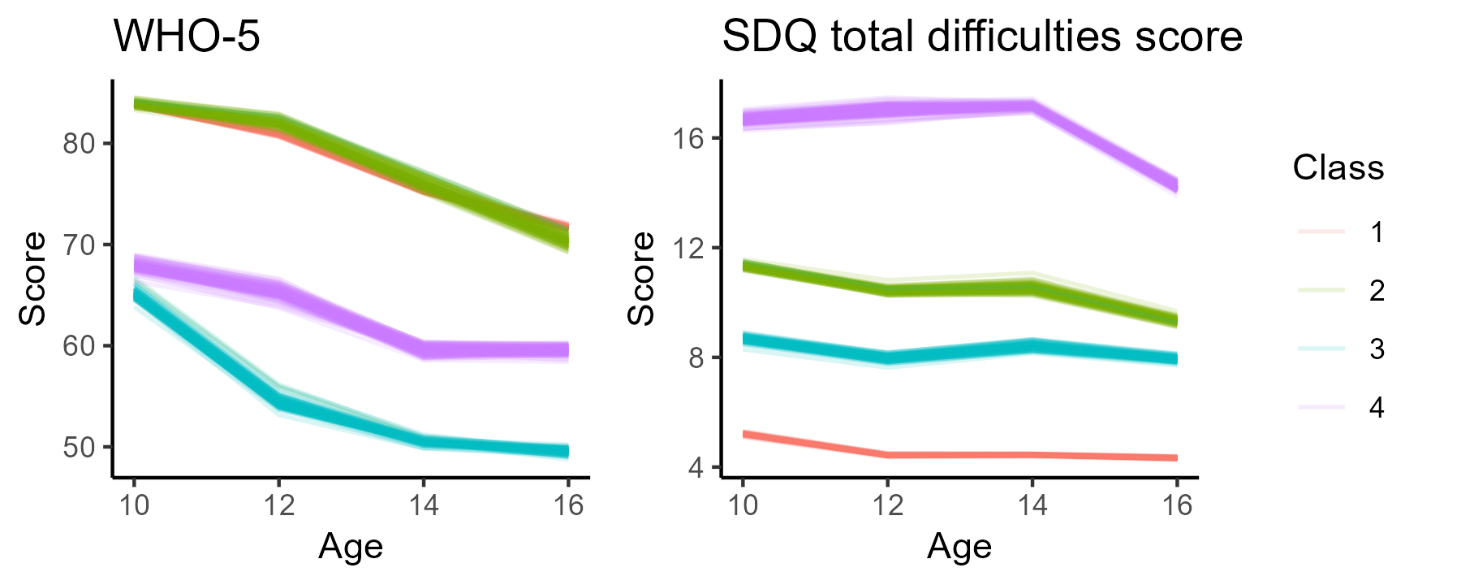
**

**Figure S5.** Results of the parallel latent class growth analysis using multiple imputations to handle missing values

We created 100 data sets thorough an imputation procedure that included all potential correlates and outcome variables. Since class assignments were probabilistic and could not be integrated as a whole, we overlaid the trajectories of each class across all 100 data sets. Class numbers were assigned in order of their proportion. The ranges of class proportions were 51.6-55.3% for class 1, 19.0-21.1% for class 2, 16.2-19.1% for class 3, and 7.4-9.3% for class 4.


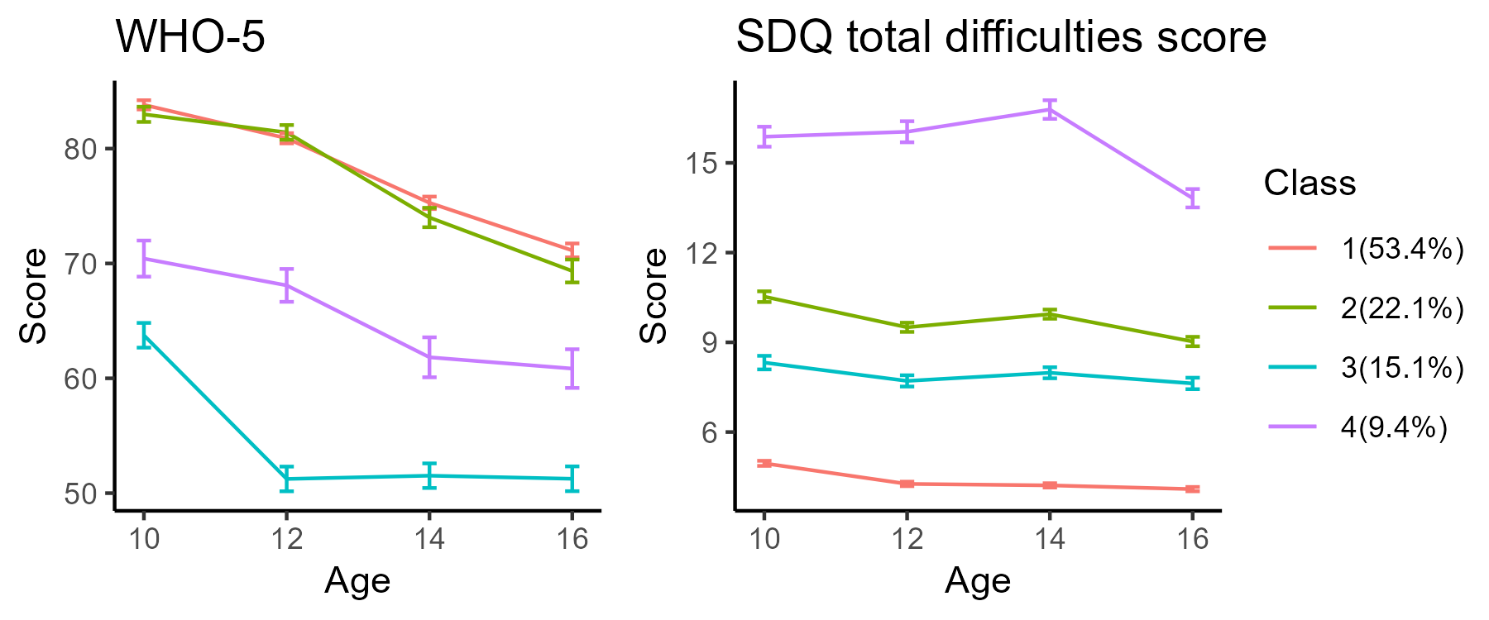


**Figure S6.** Results of the parallel latent class growth analysis in participants with complete WHO-5 and SDQ total difficulties scores

The points represent the mean of WHO-5 and SDQ total difficulties score per class. Error bars indicate standard error. Class numbers were assigned in order of proportion.


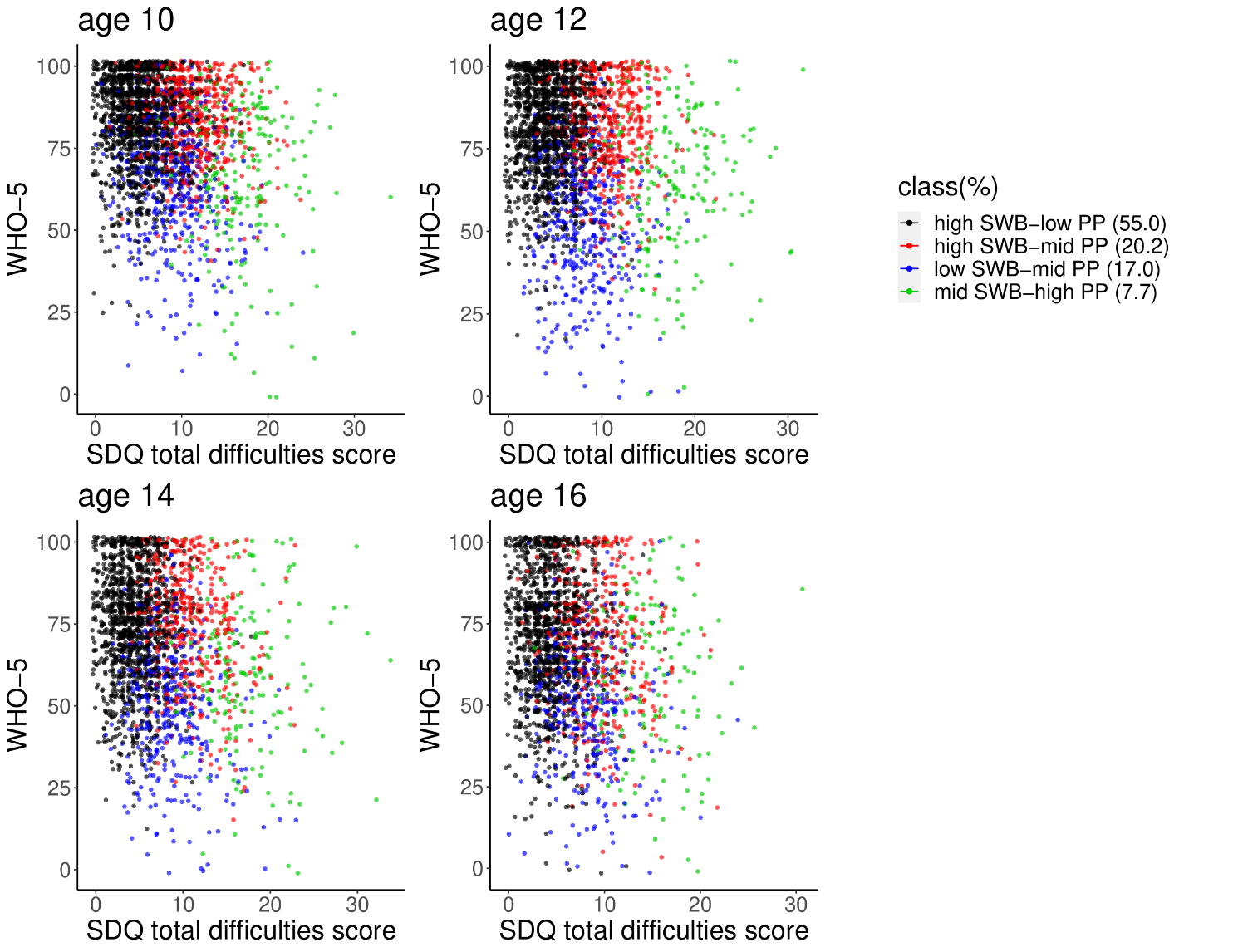


**Figure S7.** Scatter plots of WHO-5 scores and SDQ total difficulties scores at each time point

**Table S5.** Descriptive statistics of the sample population both as a whole and by classes

| **Characteristic** | **Overall N = 2994** | **high SWB-low PP N = 1648** | **high SWB-mid PP N = 606** | **low SWB-mid PP N = 508** | **Mid SWB-high PP N = 232** | ***p*-value** |
| --- | --- | --- | --- | --- | --- | --- |
| **Sociodemographic factors** |  |  |  |  |  |  |
| Female | 1407 (47.0%) | 820 (49.8%) | 256 (42.2%) | 243 (47.8%) | 88 (37.9%) | <0.001 |
| Primary caregiver |  |  |  |  |  | 0.464 |
| mother | 2949 (98.5%) | 19 (1.2%) | 14 (2.3%) | 7 (1.4%) | 3 (1.3%) |  |
| father | 43 (1.4%) | 1628 (98.8%) | 591 (97.5%) | 501 (98.6%) | 229 (98.7%) |  |
| other | 2 (0.1%) | 1 (0.1%) | 1 (0.2%) | 0 (0.0%) | 0 (0.0%) |  |
| Ethnic minority | 67 (2.4%) | 39 (2.5%) | 6 (1.1%) | 18 (3.7%) | 4 (1.8%) | 0.039 |
| missing | 150 | 73 | 39 | 23 | 15 |  |
| Annual household income | 8.1±2.6 | 8.3±2.5 | 7.8±2.7 | 8.3±2.6 | 7.3±2.6 | <0.001 |
| missing | 114 | 73 | 24 | 14 | 3 |  |
| Educational background of mother | 4.2±0.8 | 4.3±0.8 | 4.2±0.8 | 4.2±0.8 | 4.1±0.8 | <0.001 |
| missing | 22 | 9 | 8 | 2 | 3 |  |
| Educational background of father | 4.6±1.0 | 4.7±0.9 | 4.4±1.1 | 4.6±1.0 | 4.5±1.0 | <0.001 |
| missing | 139 | 67 | 35 | 25 | 12 |  |
| House ownership | 2102 (70.4%) | 1188 (72.3%) | 405 (66.9%) | 366 (72.2%) | 143 (61.9%) | 0.002 |
| missing | 7 | 4 | 1 | 1 | 1 |  |
| Employment of mother | 1758 (59.1%) | 954 (58.2%) | 370 (61.7%) | 295 (58.1%) | 139 (60.4%) | 0.464 |
| missing | 17 | 9 | 6 | 0 | 2 |  |
| Employment of father | 2809 (98.3%) | 1556 (98.5%) | 565 (98.6%) | 472 (97.5%) | 216 (98.2%) | 0.489 |
| missing | 137 | 68 | 33 | 24 | 12 |  |
| **Individual factors** |  |  |  |  |  |  |
| Pubertal stage | 1.6±0.7 | 1.5±0.7 | 1.6±0.7 | 1.6±0.7 | 1.6±0.8 | 0.579 |
| missing | 294 | 154 | 66 | 46 | 28 |  |
| Intelligence quotient (IQ) | 107.8±14.0 | 108.2±13.9 | 106.8±14.3 | 108.5±13.3 | 105.5±15.4 | 0.006 |
| missing | 3 | 0 | 2 | 0 | 1 |  |
| Help-seeking intention | 2306 (78.2%) | 1334 (81.6%) | 477 (80.2%) | 332 (67.8%) | 163 (70.9%) | <0.001 |
| missing | 45 | 14 | 11 | 18 | 2 |  |
| Aspirations | 3.3±0.8 | 3.4±0.8 | 3.3±0.8 | 3.0±0.9 | 3.0±1.0 | <0.001 |
| missing | 10 | 4 | 4 | 1 | 1 |  |
| Self-control | 5.7±1.3 | 5.8±1.3 | 5.6±1.2 | 5.7±1.2 | 5.3±1.5 | <0.001 |
| missing | 1120 | 610 | 218 | 199 | 93 |  |
| Prosocial behavior | 3.5±0.7 | 3.6±0.6 | 3.5±0.7 | 3.3±0.7 | 3.3±0.7 | <0.001 |
| missing | 16 | 8 | 3 | 3 | 2 |  |
| Experience of kindergarten entrance exam | 765 (25.8%) | 449 (27.4%) | 135 (22.5%) | 130 (26.0%) | 51 (22.2%) | 0.062 |
| missing | 28 | 12 | 6 | 8 | 2 |  |
| Experience of elementary school entrance exam | 434 (14.5%) | 265 (16.1%) | 71 (11.7%) | 78 (15.4%) | 20 (8.7%) | 0.003 |
| missing | 7 | 4 | 1 | 1 | 1 |  |
| Tutoring school | 1186 (39.7%) | 617 (37.5%) | 244 (40.3%) | 230 (45.4%) | 95 (40.9%) | 0.016 |
| missing | 4 | 3 | 0 | 1 | 0 |  |
| Chronic health problems | 314 (10.5%) | 93 (5.7%) | 88 (14.6%) | 60 (11.8%) | 73 (31.7%) | <0.001 |
| missing | 7 | 3 | 2 | 0 | 2 |  |
| Special needs | 324 (11.0%) | 88 (5.4%) | 97 (16.2%) | 63 (12.5%) | 76 (32.9%) | <0.001 |
| missing | 40 | 24 | 9 | 6 | 1 |  |
| Overweight | 175 (5.9%) | 85 (5.2%) | 40 (6.6%) | 35 (6.9%) | 15 (6.5%) | 0.359 |
| missing | 6 | 3 | 3 | 0 | 0 |  |
| **Familial factors** |  |  |  |  |  |  |
| Separation from mother | 18 (0.6%) | 6 (0.4%) | 6 (1.0%) | 1 (0.2%) | 5 (2.2%) | 0.003 |
| missing | 4 | 3 | 1 | 0 | 0 |  |
| Separation from father | 291 (9.8%) | 141 (8.6%) | 66 (11.0%) | 58 (11.4%) | 26 (11.3%) | 0.118 |
| missing | 10 | 3 | 5 | 0 | 2 |  |
| Number of siblings | 1.1±0.8 | 1.2±0.8 | 1.1±0.8 | 1.1±0.8 | 1.1±0.8 | 0.578 |
| Elder sibling | 1,308 (43.7%) | 739 (44.8%) | 245 (40.4%) | 231 (45.5%) | 93 (40.1%) | 0.105 |
| Frequency of meeting grandparent | 4.9±1.8 | 4.9±1.8 | 4.9±1.8 | 4.9±1.8 | 5.1±1.9 | 0.173 |
| missing | 38 | 19 | 12 | 5 | 2 |  |
| Smoker in household | 144 (4.9%) | 75 (4.6%) | 32 (5.4%) | 25 (5.0%) | 12 (5.3%) | 0.888 |
| missing | 41 | 20 | 8 | 9 | 4 |  |
| Life satisfaction of caregiver | 7.3±2.0 | 7.7±1.8 | 7.0±2.0 | 6.8±2.1 | 6.1±2.3 | <0.001 |
| missing | 7 | 3 | 2 | 2 | 0 |  |
| Chronic health problems of mother | 446 (15.1%) | 160 (9.8%) | 110 (18.5%) | 108 (21.4%) | 68 (29.8%) | <0.001 |
| missing | 34 | 14 | 13 | 3 | 4 |  |
| Chronic health problems of father | 338 (11.9%) | 142 (9.0%) | 77 (13.6%) | 76 (15.7%) | 43 (19.6%) | <0.001 |
| missing | 146 | 70 | 38 | 25 | 13 |  |
| Cooperative spouse in childcare | 3.2±0.7 | 3.3±0.7 | 3.2±0.7 | 3.1±0.7 | 3.0±0.8 | <0.001 |
| missing | 163 | 81 | 39 | 27 | 16 |  |
| Satisfied with family | 5.3±1.1 | 6.5±0.9 | 6.3±1.0 | 5.9±1.4 | 5.7±1.4 | <0.001 |
| missing | 53 | 31 | 12 | 6 | 4 |  |
| Frequency of talking with caregiver | 3.0±0.8 | 3.1±0.8 | 2.9±0.8 | 2.9±0.8 | 2.9±0.8 | <0.001 |
| missing | 12 | 5 | 3 | 2 | 2 |  |
| Want to be like mother | 3.1±0.9 | 3.2±0.9 | 3.1±1.0 | 2.9±0.9 | 2.9±1.0 | <0.001 |
| missing | 64 | 36 | 11 | 10 | 7 |  |
| Want to be like father | 2.9±0.9 | 3.0±0.9 | 2.9±1.0 | 2.6±1.0 | 2.6±1.0 | <0.001 |
| missing | 69 | 39 | 14 | 9 | 7 |  |
| Family involvement in future career preferences | 1.9±0.9 | 1.8±0.9 | 1.9±1.0 | 2.0±1.0 | 2.0±0.9 | 0.002 |
| missing | 4 | 1 | 0 | 3 | 0 |  |
| Caregiver life plan is stability-oriented | 4.8±2.4 | 4.6±2.4 | 4.8±2.3 | 4.9±2.4 | 5.2±2.5 | 0.003 |
| missing | 36 | 21 | 5 | 8 | 2 |  |
| **Socioenvironmental factors** |  |  |  |  |  |  |
| Like school | 2.5±0.6 | 2.6±0.5 | 2.5±0.6 | 2.2±0.6 | 2.2±0.6 | <0.001 |
| missing | 37 | 23 | 7 | 4 | 3 |  |
| Social relationships outside school | 3.2±0.8 | 3.3±0.8 | 3.2±0.9 | 3.0±0.9 | 3.0±0.9 | <0.001 |
| missing | 15 | 6 | 2 | 3 | 4 |  |
| Parks or playgrounds in neighborhood | 2870 (95.9%) | 1587 (96.4%) | 584 (96.4%) | 478 (94.3%) | 221 (95.3%) | 0.182 |
| missing | 2 | 1 | 0 | 1 | 0 |  |
| Friends or relatives live in neighborhood | 2505 (84.2%) | 1419 (86.8%) | 497 (82.4%) | 406 (80.1%) | 183 (79.2%) | <0.001 |
| missing | 19 | 14 | 3 | 1 | 1 |  |
| Good neighborhood for childcare | 4.2±0.8 | 4.3±0.7 | 4.1±0.8 | 4.1±0.8 | 3.9±0.8 | <0.001 |
| missing | 4 | 4 | 0 | 0 | 0 |  |
| Neighborhood cohesion and trust | 17.7±3.2 | 18.1±3.2 | 17.4±3.2 | 17.3±3.1 | 17.0±3.3 | <0.001 |
| missing | 14 | 7 | 3 | 2 | 2 |  |

We used the chi-square test for categorical variables and the t-test for continuous variables to compare participant characteristics between classes.

**Table S6.** Variation inflation factor (VIF) of correlates included in multinominal regression

| **Correlates** | **VIF** |
| --- | --- |
| Female | 2.7 |
| Ethnic minority | 1.2 |
| Annual household income | 1.8 |
| Educational background of mother | 1.5 |
| Educational background of father | 1.7 |
| Pubertal stage | 1.2 |
| IQ | 1.6 |
| Aspirations | 1.3 |
| Self-control | 1.6 |
| Prosocial behavior | 1.4 |
| Experience of kindergarten entrance exam | 1.6 |
| Experience of elementary school entrance exam | 1.5 |
| Tutoring school | 2.2 |
| Chronic health problems | 2.0 |
| Special needs | 2.0 |
| Overweight | 1.3 |
| Separation from mother | 1.5 |
| Separation from father | 1.5 |
| Number of siblings | 1.5 |
| Elder sibling | 2.5 |
| Frequency of meeting grandparent | 1.2 |
| Smoker in household | 1.3 |
| Life satisfaction of caregiver | 1.6 |
| Chronic health problems of mother | 1.8 |
| Chronic health problems of father | 1.6 |
| Cooperative spouse in childcare | 1.5 |
| Satisfied with family | 1.5 |
| Frequency of talking with caregiver | 1.2 |
| Want to be like mother | 1.8 |
| Want to be like father | 1.7 |
| Family involvement in future career preferences | 1.2 |
| Caregiver life plan is stability-oriented | 1.2 |
| Like school | 1.4 |
| Social relationships outside school | 1.3 |
| Good neighborhood for childcare | 1.4 |
| Neighborhood cohesion and trust | 1.4 |

The maximum VIF in the 100 data sets of the multiple imputations for each variable was shown.

**Table S7.** Full result of the multinomial logistic regression analysis to examine correlates of each class membership

|  | **high SWB-mid PP** | | | **low SWB-mid PP** | | | **mid SWB-high PP** | | |
| --- | --- | --- | --- | --- | --- | --- | --- | --- | --- |
| **Correlates** | **OR** | **95%CI** | ***p*-value** | **OR** | **95%CI** | ***p*-value** | **OR** | **95%CI** | ***p*-value** |
| Female (vs male) | 0.87 | 0.69 - 1.08 | 0.209 | 1.23 | 0.96 - 1.57 | 0.108 | 0.77 | 0.54 - 1.09 | 0.144 |
| Ethnic minority (vs no) | 0.41 | 0.17 - 0.99 | **0.048** | 1.25 | 0.66 - 2.36 | 0.487 | 0.58 | 0.18 - 1.81 | 0.345 |
| Annual household income (1 SD) | 0.99 | 0.88 - 1.12 | 0.884 | 1.2 | 1.05 - 1.38 | **0.008** | 0.97 | 0.81 - 1.18 | 0.774 |
| Educational background of mother (1 SD) | 0.95 | 0.85 - 1.07 | 0.401 | 0.95 | 0.84 - 1.08 | 0.416 | 0.91 | 0.77 - 1.09 | 0.308 |
| Educational background of father (1 SD) | 0.86 | 0.77 - 0.97 | **0.013** | 0.96 | 0.84 - 1.09 | 0.531 | 0.98 | 0.82 - 1.18 | 0.841 |
| Pubertal stage (1 SD) | 0.97 | 0.88 - 1.08 | 0.633 | 0.99 | 0.88 - 1.11 | 0.892 | 0.96 | 0.81 - 1.13 | 0.617 |
| Intelligence quotient (IQ) (1 SD) | 0.99 | 0.88 - 1.12 | 0.898 | 1.08 | 0.95 - 1.23 | 0.227 | 1.01 | 0.84 - 1.22 | 0.887 |
| Aspirations (1 SD) | 1.01 | 0.91 - 1.13 | 0.825 | 0.82 | 0.74 - 0.91 | **<0.001** | 0.85 | 0.73 - 0.99 | **0.040** |
| Self-control (1 SD) | 0.92 | 0.81 - 1.06 | 0.255 | 1 | 0.86 - 1.16 | 0.988 | 0.84 | 0.66 - 1.07 | 0.150 |
| Prosocial behavior (1 SD) | 1.04 | 0.94 - 1.16 | 0.449 | 0.86 | 0.77 - 0.96 | **0.006** | 0.89 | 0.77 - 1.04 | 0.143 |
| Experience of kindergarten entrance exam (vs no) | 0.91 | 0.72 - 1.16 | 0.456 | 0.99 | 0.76 - 1.29 | 0.951 | 1 | 0.68 - 1.46 | 0.994 |
| Experience of elementary school entrance exam (vs no) | 0.79 | 0.58 - 1.08 | 0.136 | 0.91 | 0.66 - 1.27 | 0.583 | 0.65 | 0.37 - 1.12 | 0.120 |
| Tutoring school (vs no) | 1.27 | 1.02 - 1.57 | **0.032** | 1.34 | 1.06 - 1.7 | **0.014** | 1.33 | 0.95 - 1.86 | 0.095 |
| Chronic health problems (vs no) | 1.63 | 1.14 - 2.34 | **0.007** | 1.4 | 0.93 - 2.1 | 0.107 | 2.81 | 1.79 - 4.42 | **0.000** |
| Special needs (vs no) | 2.45 | 1.74 - 3.45 | **<0.001** | 1.78 | 1.2 - 2.65 | **0.004** | 4.3 | 2.78 - 6.65 | **0.000** |
| Overweight (vs no) | 1.02 | 0.68 - 1.54 | 0.912 | 1.06 | 0.67 - 1.66 | 0.801 | 0.75 | 0.39 - 1.44 | 0.389 |
| Separation from mother (vs no) | 1.46 | 0.43 - 4.96 | 0.546 | 0.25 | 0.03 - 2.21 | 0.213 | 2.63 | 0.67 - 10.31 | 0.166 |
| Separation from father (vs no) | 1.05 | 0.75 - 1.47 | 0.790 | 1.07 | 0.75 - 1.54 | 0.714 | 0.83 | 0.49 - 1.39 | 0.471 |
| Number of siblings (1 SD) | 0.94 | 0.84 - 1.05 | 0.284 | 0.98 | 0.86 - 1.11 | 0.736 | 1 | 0.84 - 1.19 | 0.989 |
| Elder sibling (vs no) | 0.77 | 0.62 - 0.97 | **0.024** | 0.83 | 0.65 - 1.07 | 0.151 | 0.64 | 0.45 - 0.92 | **0.015** |
| Frequency of meeting grandparent (1 SD) | 1.04 | 0.94 - 1.15 | 0.451 | 1.06 | 0.95 - 1.19 | 0.291 | 1.26 | 1.08 - 1.47 | **0.003** |
| Smoker in household (vs no) | 0.87 | 0.55 - 1.38 | 0.549 | 1.12 | 0.67 - 1.89 | 0.669 | 0.84 | 0.4 - 1.74 | 0.634 |
| Life satisfaction of caregiver (1 SD) | 0.85 | 0.76 - 0.96 | **0.007** | 0.76 | 0.68 - 0.86 | **<0.001** | 0.62 | 0.53 - 0.73 | **<0.001** |
| Chronic health problems of mother | 1.53 | 1.14 - 2.05 | **0.005** | 1.89 | 1.39 - 2.57 | **<0.001** | 2.04 | 1.37 - 3.04 | **<0.001** |
| Chronic health problems of father | 1.1 | 0.8 - 1.52 | 0.567 | 1.24 | 0.88 - 1.74 | 0.212 | 1.14 | 0.73 - 1.78 | 0.554 |
| Cooperative spouse in childcare (1 SD) | 0.85 | 0.76 - 0.94 | **0.003** | 0.89 | 0.79 – 1 | **0.045** | 0.96 | 0.81 - 1.13 | 0.602 |
| Satisfied with family (1 SD) | 0.91 | 0.81 - 1.02 | 0.101 | 0.76 | 0.68 - 0.85 | **<0.001** | 0.68 | 0.59 - 0.79 | **<0.001** |
| Frequency of talking with caregiver (1 SD) | 0.83 | 0.75 - 0.91 | **<0.001** | 0.87 | 0.78 - 0.98 | **0.017** | 0.9 | 0.77 - 1.05 | 0.179 |
| Want to be like mother (1 SD) | 0.87 | 0.77 - 0.98 | **0.026** | 0.88 | 0.77 - 1.01 | 0.065 | 0.94 | 0.78 - 1.13 | 0.501 |
| Want to be like father (1 SD) | 1.1 | 0.97 - 1.24 | 0.123 | 0.91 | 0.8 - 1.04 | 0.169 | 0.89 | 0.74 - 1.06 | 0.181 |
| Family involvement in future career preferences (1 SD) | 1.1 | 0.99 - 1.21 | 0.072 | 1.14 | 1.02 - 1.27 | **0.021** | 1.19 | 1.02 - 1.39 | **0.025** |
| Caregiver life plan is stability-oriented (1 SD) | 1.03 | 0.93 - 1.14 | 0.613 | 1.06 | 0.95 - 1.18 | 0.317 | 1.09 | 0.93 - 1.28 | 0.266 |
| Like school (1 SD) | 0.84 | 0.75 - 0.94 | **0.002** | 0.62 | 0.55 - 0.7 | **<0.001** | 0.67 | 0.57 - 0.78 | **<0.001** |
| Social relationships outside school (1 SD) | 0.89 | 0.8 - 0.99 | **0.026** | 0.76 | 0.68 - 0.85 | **<0.001** | 0.7 | 0.6 - 0.81 | **<0.001** |
| Good neighborhood for childcare (1 SD) | 0.88 | 0.79 - 0.98 | **0.016** | 0.93 | 0.83 - 1.05 | 0.228 | 0.84 | 0.72 - 0.98 | **0.030** |
| Neighborhood cohesion and trust (1 SD) | 0.96 | 0.87 - 1.07 | 0.496 | 0.95 | 0.84 - 1.07 | 0.406 | 1.04 | 0.88 - 1.23 | 0.642 |

**Table S8.** Full result of the pairwise logistic regression analysis examining correlates of membership in high SWB-mid PP or low SWB-mid PP

| **Characteristic** | **OR** | **95%CI** | **p-value** |
| --- | --- | --- | --- |
| Female (vs male) | 1.42 | 1.07 - 1.88 | **0.015** |
| Ethnic minority (vs no) | 3.06 | 1.19 - 7.88 | **0.021** |
| Annual household income (1 SD) | 1.22 | 1.04 - 1.42 | **0.013** |
| Educational background of mother (1 SD) | 1 | 0.87 - 1.15 | 0.977 |
| Educational background of father (1 SD) | 1.11 | 0.96 - 1.28 | 0.179 |
| Pubertal stage (1 SD) | 1.02 | 0.9 - 1.16 | 0.748 |
| IQ (1 SD) | 1.09 | 0.94 - 1.26 | 0.259 |
| Aspirations (1 SD) | 0.81 | 0.71 - 0.92 | **0.001** |
| Self-control (1 SD) | 1.09 | 0.92 - 1.3 | 0.330 |
| Prosocial behavior (1 SD) | 0.82 | 0.73 - 0.93 | **0.002** |
| Experience of kindergarten entrance exam (vs no) | 1.09 | 0.8 - 1.47 | 0.588 |
| Experience of elementary school entrance exam (vs no) | 1.16 | 0.78 - 1.71 | 0.463 |
| Tutoring school (vs no) | 1.06 | 0.81 - 1.39 | 0.652 |
| Chronic health problems (vs no) | 0.86 | 0.57 - 1.29 | 0.458 |
| Special needs (vs no) | 0.73 | 0.49 - 1.08 | 0.113 |
| Overweight (vs no) | 1.04 | 0.63 - 1.71 | 0.882 |
| Separation from mother (vs no) | 0.17 | 0.02 - 1.5 | 0.111 |
| Separation from father (vs no) | 1.02 | 0.68 - 1.53 | 0.906 |
| Number of siblings (1 SD) | 1.04 | 0.9 - 1.2 | 0.580 |
| Elder sibling (vs no) | 1.08 | 0.81 - 1.43 | 0.594 |
| Frequency of meeting grandparent (1 SD) | 1.02 | 0.9 - 1.16 | 0.744 |
| Smoker in household (vs no) | 1.28 | 0.72 - 2.29 | 0.397 |
| Life satisfaction of caregiver (1 SD) | 0.89 | 0.78 - 1.02 | 0.100 |
| Chronic health problems of mother | 1.24 | 0.89 - 1.72 | 0.203 |
| Chronic health problems of father | 1.13 | 0.78 - 1.63 | 0.527 |
| Cooperative spouse in childcare (1 SD) | 1.05 | 0.92 - 1.19 | 0.513 |
| Satisfied with family (1 SD) | 0.84 | 0.74 - 0.95 | **0.006** |
| Frequency of talking with caregiver (1 SD) | 1.06 | 0.93 - 1.2 | 0.397 |
| Want to be like mother (1 SD) | 1.01 | 0.87 - 1.17 | 0.867 |
| Want to be like father (1 SD) | 0.83 | 0.72 - 0.96 | **0.013** |
| Family involvement in future career preferences (1 SD) | 1.04 | 0.92 - 1.17 | 0.544 |
| Caregiver life plan is stability-oriented (1 SD) | 1.03 | 0.91 - 1.17 | 0.647 |
| Like school (1 SD) | 0.74 | 0.65 - 0.84 | **<0.001** |
| Social relationships outside school (1 SD) | 0.85 | 0.75 - 0.97 | **0.013** |
| Good neighborhood for childcare (1 SD) | 1.06 | 0.93 - 1.21 | 0.393 |
| Neighborhood cohesion and trust (1 SD) | 0.99 | 0.87 - 1.13 | 0.862 |

OR>1 correlates with membership to low SWB-mid PP and OR<1 correlates with membership to high SWB-mid PP.

**References**

[1.    Tanner JM. 1 Normal growth and techniques of growth assessment. *Clin Endocrinol Metab*. 1986;15(3):411-451.](https://sciwheel.com/work/bibliography/7359540)

[2.    Okada N, Yahata N, Koshiyama D, et al. Smaller anterior subgenual cingulate volume mediates the effect of girls’ early sexual maturation on negative psychobehavioral outcome. *Neuroimage*. 2020;209:116478.](https://sciwheel.com/work/bibliography/12593255)

[3.    Inada N, Kamio Y. Short Forms of the Japanese Version WISC-III for Assessment of Children with Autism Spectrum Disorders.](https://sciwheel.com/work/bibliography/15772445) *Jpn. J. Child Adolesc. Psychiatr.* 2010; 51(Supplement): 11-19.

[4.    Kanata S, Koike S, Ando S, et al. Enuresis and Hyperactivity-Inattention in Early Adolescence: Findings from a Population-Based Survey in Tokyo (Tokyo Early Adolescence Survey). *PLoS ONE*. 2016;11(7):e0158786.](https://sciwheel.com/work/bibliography/11066107)

[5.    Ando S, Usami S, Matsubayashi T, et al. Age relative to school class peers and emotional well-being in 10-year-olds. *PLoS ONE*. 2019;14(3):e0214359.](https://sciwheel.com/work/bibliography/11530982)

[6.    Rutter M, Tizard J, Whitmore K, eds. Education, Health and Behaviour. Longman Publishing Group; 1970.](https://sciwheel.com/work/bibliography/15371460)

[7.    Xu MK, Jones PB, Barnett JH, et al. Adolescent self-organization predicts midlife memory in a prospective birth cohort study. *Psychol Aging*. 2013;28(4):958-968.](https://sciwheel.com/work/bibliography/16509574)

[8.    Nishida A, Xu KM, Croudace T, Jones PB, Barnett J, Richards M. Adolescent self-control predicts midlife hallucinatory experiences: 40-year follow-up of a national birth cohort. *Schizophr Bull*. 2014;40(6):1543-1551.](https://sciwheel.com/work/bibliography/16500808)

9.    Japan Society of School Health. Manual for the medical examination of pupils. 2013. Accessed October 30, 2025 https://www.gakkohoken.jp/book/ebook/ebook_H270030/index_h5.html#%E8%A1%A8%E7%B4%99

[10.   Sampson RJ, Raudenbush SW, Earls F. Neighborhoods and violent crime: A multilevel study of collective efficacy. *Science*. 1997;277(5328):918-924.](https://sciwheel.com/work/bibliography/743318)
